# Hypoxia-Associated Alternative Polyadenylation of CARM1 and Tumor Microenvironment Alterations in Non-Small Cell Lung Cancer

**DOI:** 10.3390/genes17050505

**Published:** 2026-04-24

**Authors:** Xinyu Qin, Chunlong Zhang, Sijia Wu, Jing Lu, Guohua Wang, Yang Li

**Affiliations:** 1 School of Computer Science and Artificial Intelligence, Northeast Forestry University, Harbin 150040, China; 2 School of Life Science and Technology, Xidian University, No. 2, South Taibai Road, Yanta District, Xi’an 710071, China; 3 School of Computer Science and Technology, Harbin Institute of Technology, Harbin 150001, China

**Keywords:** non-small cell lung cancer, hypoxia, alternative polyadenylation, microRNA, cisplatin

## Abstract

Background: Tumor hypoxia in non-small cell lung cancer (NSCLC) promotes malignant progression and treatment resistance by enhancing abnormal vasculature, invasiveness, and metastasis. However, the molecular mechanisms underlying hypoxia-driven tumor progression remain incompletely understood. Methods: In this study, patient samples, cell lines, single-cell transcriptomic data, and spatial transcriptomic data were comprehensively analyzed to investigate hypoxia-associated molecular alterations in NSCLC. Results: A global trend toward shortened 3’ untranslated regions (3’UTRs) was observed in hypoxic tumors. Analysis of hypoxia-related alternative polyadenylation (APA) events revealed preferential usage of proximal polyadenylation sites (poly(A) sites, PASs) in *CARM1*. Shortening of the *CARM1* 3’UTR was associated with hypoxia and may serve as a candidate biomarker. This APA event may reduce putative microRNA (miRNA) binding sites and contribute to increased *CARM1* expression, while potentially influencing the expression of hypoxia-related genes such as *SELENBP1*. Drug sensitivity analysis further suggested that patients with shorter *CARM1* 3’UTRs may exhibit differential responses to cisplatin chemotherapy. Moreover, single-cell and spatial transcriptomic analyses demonstrated enhanced interactions between hypoxic tumor cells and fibroblasts, highlighting a potential role for APA in remodeling the hypoxic tumor microenvironment. Conclusions: Our findings identify hypoxia-related APA features and characterize hypoxia-associated alterations within the NSCLC tumor microenvironmen, providing new insights into the molecular landscape of hypoxia-associated tumor progression.

## 1. Introduction

Lung cancer remains the leading cause of cancer-related mortality worldwide [1]. Non-small cell lung cancer (NSCLC) is the predominant histological subtype, accounting for approximately 85% of all lung cancer cases [2]. Tumor hypoxia is a common feature of NSCLC, with detectable hypoxia reported in 50–80% of patients with stage I–IV disease [3]. Hypoxia promotes multiple malignant phenotypes, including proliferation, migration, invasion, and epithelial–mesenchymal transition (EMT), and is also associated with resistance to chemotherapy and immunotherapy [4]. Therefore, elucidating the molecular basis of hypoxia in NSCLC is important for understanding tumor progression and improving therapeutic strategies.

Previous studies have identified several molecular mechanisms involved in tumor hypoxia. Hypoxia-inducible factors (HIFs), particularly HIF-1α and HIF-2α, are central regulators of the hypoxic response and contribute to tumor growth, angiogenesis, invasion, and therapeutic resistance [5]. In addition, hypoxia can promote EMT by modulating EMT signaling pathways, activating EMT-related transcription factors, and reshaping miRNA regulatory networks, thereby enhancing tumor invasiveness and metastatic potential [6]. Hypoxia-associated metabolic remodeling and extracellular matrix alterations further contribute to aggressive tumor behavior and microenvironmental adaptation [7,8]. Despite these advances, most previous studies of hypoxia in lung cancer have focused on transcriptional regulation, miRNA networks, signaling pathways, or protein-level changes, whereas post-transcriptional regulation mediated by APA remains insufficiently characterized.

APA is an important post-transcriptional regulatory process that generates transcript isoforms with different 3′ untranslated region (3′UTR) lengths through the selection of alternative poly(A) sites [9,10,11]. By altering 3′UTR length, APA can influence the inclusion or exclusion of cis-regulatory elements, including miRNA-binding sites and RNA-binding protein (RBP)-binding sites, thereby affecting mRNA stability, translation, and expression [12,13,14]. Growing evidence suggests that APA may participate in tumor adaptation to hypoxia. For example, *CSTF2* has been reported to promote hepatocellular carcinoma survival under hypoxic conditions by shortening the 3′UTR of *PGK1* and enhancing glycolysis [15,16,17]. However, the role of hypoxia-associated APA in NSCLC has not been systematically investigated.

In particular, whether hypoxia-associated APA contributes to transcriptomic remodeling, tumor microenvironment alterations, and heterogeneity in therapeutic response in NSCLC remains unclear [4,18]. Although previous studies have mainly focused on hypoxia-related transcriptional programs, signaling pathways, and miRNA-mediated regulation, the landscape and functional significance of APA under hypoxic conditions in NSCLC remain largely unexplored [18,19]. Moreover, while *CARM1* has been implicated in cancer progression, transcriptional regulation, and metabolic adaptation across multiple cancer types, including NSCLC [20,21], its hypoxia-associated regulation through APA has not been reported. To our knowledge, the potential role of hypoxia-associated APA of *CARM1* in tumor microenvironmental changes and drug response has not yet been systematically investigated in NSCLC [19,22].

Here, we performed an integrative analysis of APA alterations and microenvironmental reprogramming between hypoxic and normoxic NSCLC phenotypes. By combining bulk RNA-seq data from patients and cell lines with single-cell and spatial transcriptomic datasets, we observed a global shift toward proximal poly(A) site usage under hypoxic conditions. We further identified a 12-event hypoxia-associated APA signature and constructed a hypoxia signature score (HSS) to characterize hypoxia-related APA patterns. In addition, we explored the potential associations of APA events with post-transcriptional regulation, spatial microenvironmental remodeling, and predicted drug response. Our findings provide a multi-layered view of hypoxia-associated APA in NSCLC and highlight proximal APA of *CARM1* as a candidate feature associated with hypoxic status, tumor progression, and cisplatin sensitivity.

## 2. Materials and Methods

### 2.1. Bulk and CCLE Sample Preparation

To study the effects of APA biomarkers on hypoxia, we collected data from 1013 NSCLC patients and 110 normal samples from The Cancer Genome Atlas (TCGA), as well as data from 123 NSCLC cell lines from the Cancer Cell Line Encyclopedia (CCLE) [23]. In addition to expression data, clinical information for the 1013 patients was also included, such as overall survival, tumor stage, and treatment data. These data were used to evaluate the clinical relevance of APA events and gene expression biomarkers.

### 2.2. Detection of APA Events and Gene Expression Quantification

APA events for TCGA patients were directly downloaded from The Cancer 3’UTR Atlas (TC3A). To detect APA events in the 123 CCLE samples, we followed a procedure similar to that used in TC3A [24]. Briefly, Samtools (version 1.9) was used to extract sequences and quality information from RNA-seq BAM files. STAR (version 2.7.9a) [25] was then used to align the reads to the hg19 reference genome (GENCODE v37lift37). DaPars [26] was applied to identify APA events and to quantify the percentage of distal poly(A) site usage index (PDUI) for each transcript. APA events with less than 60% missing values and less than 40% constant PDUI values (equal to 1 or 0) were defined as informative APA events. In total, 6914 informative APA events in patients and 16,259 informative APA events in cell lines were retained for subsequent analysis.

Gene expression data for patients were downloaded from TCGA (hg19). For cell lines, gene expression data (upper quartile normalized, UQN) were processed using the same reference genome and a similar pipeline as TCGA, with RSEM. Genes with low expression (average expression < 1 UQN) were excluded from further analysis. After filtering, 17,735 informative genes in patients and 7899 informative genes in cell lines were retained.

### 2.3. Definition of Hypoxia and Normoxia Status

To classify hypoxic and normoxic phenotypes in NSCLC patients and cell lines, two complementary approaches were used to integrate prior biological knowledge of hypoxia with data-driven transcriptomic structure. First, hypoxia scores were calculated for 1013 patients and 123 cell lines with mRNA expression data using three previously reported mRNA-based hypoxia signatures developed by Winter et al. [27], Buffa et al. [28], and Ragnum et al. [29]. For each gene in each signature, samples with expression values in the top 50% were assigned a score of +1, whereas those in the bottom 50% were assigned a score of −1. Gene-wise scores were summed across all genes in each signature to generate a hypoxia score for each sample. Samples with scores greater than 0 were classified as hypoxic, those with scores less than 0 were classified as normoxic, and those with scores equal to 0 were classified as mixed [30]. This approach was referred to as the NC method and represented a signature-guided classification strategy anchored to established hypoxia-responsive transcriptional programs (Figure S1A).

Second, non-negative matrix factorization (NMF) consensus clustering was performed on gene expression matrices from patients and cell lines. Cluster-level hypoxia enrichment was evaluated using Gene Set Variation Analysis (GSVA) [31] with the HYPOXIA gene set from the Molecular Signatures Database (MSigDB) [32]. The cluster with the highest hypoxia enrichment score was defined as hypoxic, the cluster with the lowest score was defined as normoxic, and the remaining clusters were classified as mixed [33]. This approach was referred to as the NMF method and represented a data-driven unsupervised classification strategy that captured intrinsic transcriptomic structure and heterogeneity among samples.

To improve the robustness of phenotype assignment, the classifications generated by the NC and NMF methods were integrated. Samples that were concordantly classified as hypoxic or normoxic by both methods were assigned to the corresponding group, whereas all remaining samples were categorized as mixed. This consensus framework was adopted to define high-confidence hypoxic and normoxic samples by integrating complementary evidence from a signature-guided method and a data-driven method, thereby reducing potential misclassification and limiting dependence on any single algorithmic assumption. Sample distributions were visualized using principal component analysis (PCA) [34]. The reliability of the classification was further evaluated using GSVA with the HYPOXIA, EPITHELIAL_MESENCHYMAL_TRANSITION, and GLYCOLYSIS gene sets from MSigDB [32]. The hypoxic microenvironment is a key inducer of EMT-related gene expression [6]. Hypoxia upregulates glycolytic genes through the activation of hypoxia-inducible factors (HIF), promoting a shift from oxidative phosphorylation to glycolysis to adapt to the low-oxygen environment [35]. Gene enrichment analysis was performed using DAVID [36,37].

### 2.4. Identification of Hypoxia-Related APA Biomarkers

Tumor hypoxia-related APA biomarkers were first identified as candidate events showing significantly altered poly(A) site usage in hypoxic tumors (nominal P < 0.010 and |log2FC| > 0.050), together with significant associations with hypoxia-related genes (nominal P < 0.010 and |R| > 0.1). These thresholds were selected to balance statistical rigor and sensitivity for detecting subtle but potentially biologically meaningful APA alterations in heterogeneous tumor transcriptomic data.

Hypoxia-related genes were identified by integrating three lines of evidence: differential expression between hypoxic and normoxic groups, differential expression between tumor and normal samples, and prior literature support for functional relevance in lung cancer (Table S1). For differential expression analysis, the same thresholds (nominal P < 0.010 and |log2FC| > 0.050) were applied. For genome-wide exploratory analyses, nominal P value thresholds were used in combination with effect-size filters to define candidate APA events and genes. For enrichment analyses involving multiple testing, false discovery rate (FDR) correction was applied where appropriate.

Candidate APA events were then entered into logistic regression analysis in the TCGA dataset to identify biomarkers independently associated with tumor hypoxia. TCGA was used for model development and internal validation, whereas the CCLE dataset was used as an independent validation cohort. The hypoxia signature score (HSS) was calculated as a linear combination of selected APA biomarkers weighted by their corresponding regression coefficients, with higher HSS values indicating a greater degree of tumor hypoxia. The HSS of the k-th sample was defined as follows:

$$HSS=\sum_{k}Coefficient\left(APA\_event_{k}\right)\times PDUI\left(APA\_event_{k}\right)$$

The performance of the HSS was evaluated at multiple levels. Discriminative ability was assessed using the AUC and by comparing HSS values between predefined hypoxic and normoxic samples. Biological consistency was evaluated by examining the correlation between HSS and hypoxia enrichment scores. Clinical relevance was assessed through survival differences between high and low HSS groups and associations with drug response-related phenotypes. In addition, the generalizability of the HSS was evaluated in the independent CCLE dataset. The overall workflow for the integrated analysis of TCGA and CCLE RNA-seq data are summarized in Figure S11.

### 2.5. Analysis of APA-Associated miRNA Regulation During Tumor Hypoxia

The selection of alternative poly(A) sites alters the length of 3’UTRs and may affect miRNA-mediated gene regulation. According to TargetScan (version 7.2) [38], the use of proximal poly(A) sites is associated with the loss of predicted conserved miRNA-binding sites. We observed that PDUI values of APA events were negatively correlated with the expression of their corresponding genes, suggesting a potential link between APA and post-transcriptional regulation. In addition, proximal poly(A) site usage was associated with increased expression of APA-regulated genes. This pattern may also influence the availability of miRNAs and their interactions with other target genes (referred to here as competing genes). These associations were supported by coordinated expression patterns, including positive correlations among certain gene pairs and inverse correlations with PDUI values.

### 2.6. Drug Sensitivity Analysis

APA events may influence the ability of tumor cells to adapt to hypoxic conditions and may be associated with variation in drug response. Potential drugs were first identified from the Comparative Toxicogenomics Database (CTD) [39] based on genes associated with APA biomarkers. These drugs were then intersected with clinically used medications in NSCLC patients from TCGA. Drug sensitivity was evaluated using pRRophetic [40]. Correlation analysis showed that PDUI values were positively associated with the half-maximal inhibitory concentration (IC50). This result suggests that tumor samples with lower PDUI values (shorter 3’UTRs) may be associated with increased drug sensitivity. Differences in predicted drug sensitivity were further compared between APA-defined groups. These analyses suggest that APA patterns may be associated with variability in drug response. To further explore clinical relevance, survival outcomes were compared between patients with different PDUI levels under corresponding drug treatments in the TCGA cohort. These results suggest that APA events may have potential value for guiding treatment stratification. However, these findings are based on predictive modeling and retrospective analyses and should be interpreted with caution.

### 2.7. Single-Cell Analysis of Hypoxic and Normoxic Malignant Cells

The single-cell RNA sequencing (scRNA-seq) data of 24 NSCLC patients were downloaded from the ArrayExpress database (E-MTAB-6149 and E-MTAB-6653) [41]. Raw FASTQ files were processed using CellRanger count (version 4.0.0) and aligned to the human reference genome hg19 with default settings. Because these datasets were generated using a 3′ end-capture scRNA-seq workflow, they were compatible with APA analysis focused on poly(A) site usage within 3′ untranslated regions (3′-UTRs). After integration of the scRNA-seq data, quality control was performed using Seurat (version 5.0.1). Only cells with 101–6000 detected genes, more than 200 UMIs (unique molecular identifiers), and less than 10% mitochondrial UMIs were retained for downstream analyses. Cell types were annotated using marker genes reported in the previous study [41]. To identify hypoxic and normoxic malignant cells, inferCNV [42] was first applied to infer chromosome-scale or arm-scale CNV patterns in malignant cells, using fibroblasts, epithelial cells, and endothelial cells as reference populations. The inferred malignant cells were then further classified by integrating hypoxia-related gene set scores calculated using ssGSEA [43] and pseudotime trajectories inferred using Monocle2 [44]. In addition, CIBERSORTx [45] was used for deconvolution of bulk transcriptome data, CellPhoneDB (version 5.0.1) [46] was used to analyze cell–cell interactions, and pySCENIC (version 0.12.1) [47] was applied to identify key transcription factors in cells under hypoxic and normoxic conditions.

To quantify APA at single-cell resolution, aligned reads for each cell were extracted from the integrated BAM file using SplitBam [48]. The resulting BAM files were converted into bedgraph format using bedtools, and APA events within 3′-UTRs were detected using DaPars to calculate PDUI values for individual cells. Considering the sparse coverage inherent to scRNA-seq data, downstream analyses emphasized group-level APA usage trends and PDUI distribution differences between hypoxic and normoxic cells, rather than isolated APA calls from individual cells. To improve robustness, only APA events detected in more than 20 cells in both the hypoxic and normoxic groups were retained for differential analysis. Furthermore, the major APA patterns identified from scRNA-seq data were compared with bulk RNA-seq results to assess cross-platform consistency. The overall workflow of the single-cell classification and APA analysis is summarized in Figure S12.

## 3. Results

### 3.1. Shortened 3’UTRs Length in Hypoxic Tumors of NSCLC

**Figure 1:**
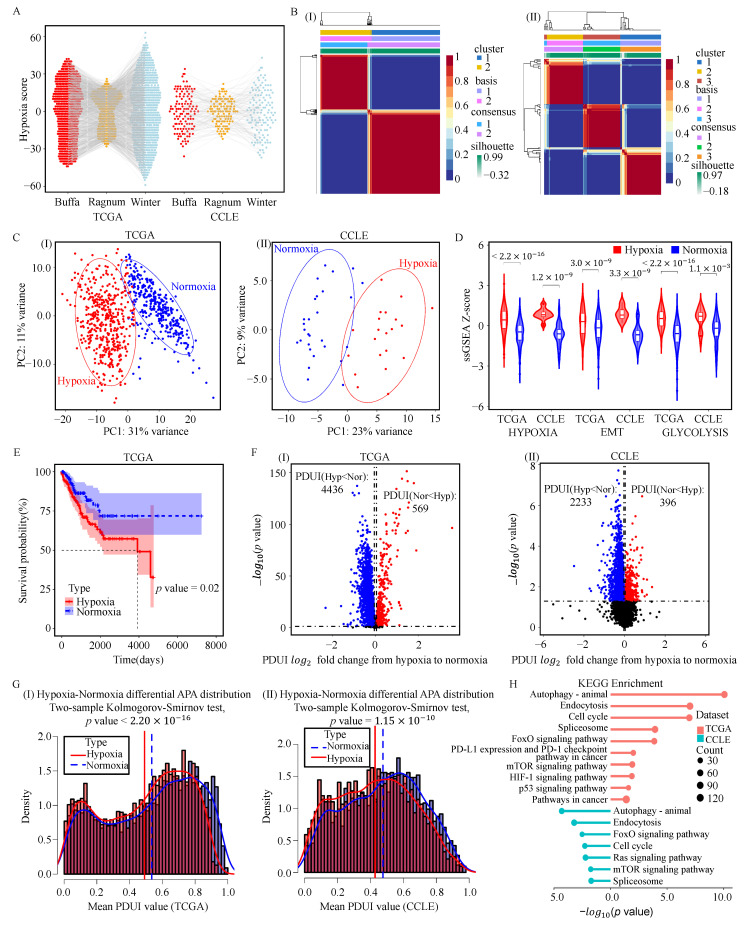
Overview of APA alterations in hypoxic NSCLC samples. (**A**) Hypoxia scores of 1013 NSCLC patients from TCGA and 123 NSCLC cell lines from CCLE were calculated using the Buffa, Winter, and Ragnum mRNA abundance-based hypoxia signatures. (**B**) NMF consensus clustering of 1013 TCGA patients (**I**) and 123 CCLE cell lines (**II**). Samples were classified as hypoxic or normoxic according to the integrated NC and NMF framework described in Methods; samples not concordantly classified by both methods were assigned to the mixed group. (**C**) PCA plots showing the separation of hypoxic and normoxic samples in TCGA patients (**I**) and CCLE cell lines (**II**) based on hypoxia-related gene expression profiles. (**D**) Comparison of hypoxia-related gene set scores between the defined hypoxic and normoxic groups by the Wilcoxon rank-sum test. (**E**) Kaplan–Meier survival analysis comparing overall survival between hypoxic and normoxic patients in the TCGA cohort; significance was assessed by the log-rank test. (**F**) Differential APA events between hypoxic and normoxic patients (**I**) and cell lines (**II**), identified using nominal *P* < 0.010 and |log2FC| > 0.050 by Student’s *t*-test. In hypoxic patients, 4436 APA events showed lower PDUI values and 569 showed higher PDUI values. In hypoxic cell lines, 2233 APA events showed lower PDUI values and 396 showed higher PDUI values. (**G**) The average PDUI distributions of differential APA events for hypoxic and normoxic patients (**I**) and cell lines (**II**). (**H**) KEGG pathways enriched for genes with differential APA events in TCGA patients (**top**) and CCLE cell lines (**bottom**), identified using DAVID; multiple-testing correction was applied where appropriate for enrichment analysis.

Hypoxic scores for 1013 NSCLC patients and 123 NSCLC cell lines were obtained separately using the NC method [30] (Figure 1A and Figure S2A). The Non-negative Matrix Factorization (NMF) method (see Methods) was used to cluster 1013 NSCLC patients into two groups and 123 NSCLC cell lines into three groups (Figure 1B and Figure S2B,C). Our pipeline (see Methods) identified 443 hypoxic and 344 normoxic patients in TCGA, as well as 21 hypoxic and 28 normoxic cell lines in CCLE (Figure S1B). The two groups were clearly separated using the first two principal components of the expression of hypoxia-related gene sets (Figure 1C). Compared to normoxic samples, hypoxic samples showed higher scores in three hypoxia-related pathways (Figure 1D), and the expression level of the *HIF1A* gene was also higher in hypoxic samples (Figure S2D). The results from patients and cell lines showed consistent separation patterns, supporting the validity of our workflow for distinguishing hypoxic and normoxic phenotypes.

Based on this classification, survival outcomes were compared between hypoxic and normoxic patients. Hypoxic patients exhibited significantly poorer overall survival than normoxic patients (Figure 1E). We next compared the PDUI of informative APA events between the two groups. In hypoxic patients, 4436 transcripts exhibited significantly lower PDUI values, exceeding the number of transcripts with increased PDUI values (Figure 1F). Similarly, in hypoxic cell lines, 2233 transcripts showed reduced PDUI values, also exceeding those with the opposite trend (Figure 1F). The overall PDUI distribution further indicated that hypoxic samples had significantly lower PDUI values (Figure 1G), suggesting a global shift toward shorter 3’UTRs under hypoxic conditions.

These differential APA events involved 4739 genes in TCGA patients and 2537 genes in CCLE cell lines. Functional enrichment analysis showed that these genes were significantly associated with hypoxia-related pathways (Figure 1H and Figure S2E). For example, the HIF-1 signaling pathway has been reported to contribute to tumor progression in NSCLC by regulating glycolysis, angiogenesis, and cell cycle in response to hypoxia [49,50]. The mTOR signaling pathway has also been implicated in gene expression regulation, metabolism, and cell survival under hypoxic conditions [51]. In addition, oxidative stress and hypoxia are interconnected features of the tumor microenvironment and have been associated with processes such as angiogenesis, invasion, metabolic reprogramming, and immune evasion [52]. These pathway associations suggest that APA alterations may be linked to hypoxia-related biological processes in NSCLC.

**Figure 2:**
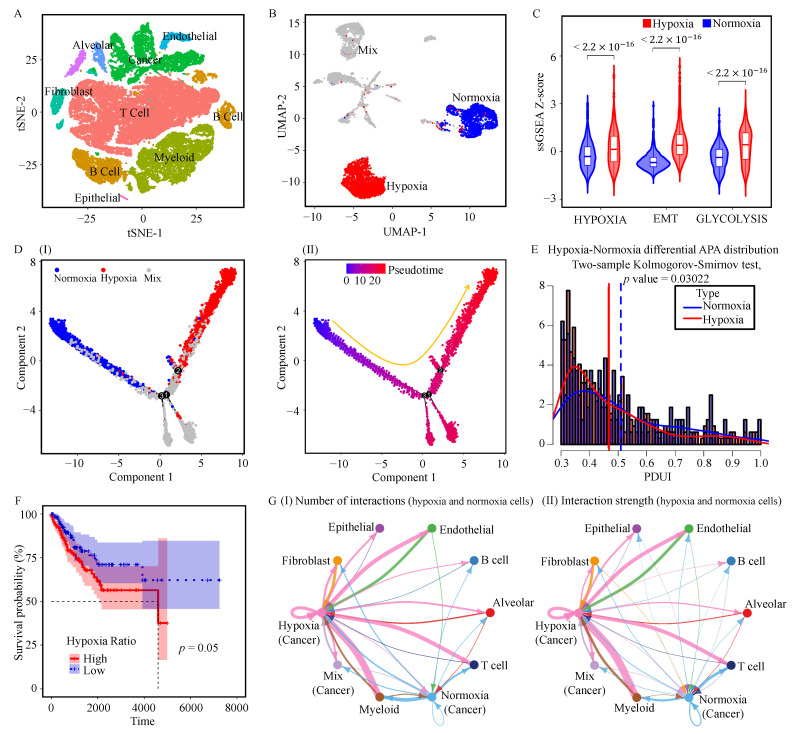
Single-cell analysis of hypoxic and normoxic tumor cells in NSCLC. (**A**) Annotation of eight major cell types in the scRNA-seq dataset from 24 NSCLC patients. Cell type annotation was performed based on published marker genes as described in Methods. (**B**) UMAP visualization of hypoxic and normoxic malignant cells. Hypoxic and normoxic cells were defined by integrating inferCNV results with ssGSEA-based hypoxia scores; cells not confidently assigned to either group were classified as mixed cells. (**C**) Comparison of hypoxia-related gene set scores between hypoxic and normoxic cells by the Wilcoxon rank-sum test. (**D**) Pseudotime trajectory of hypoxic and normoxic malignant cells inferred with Monocle2, colored according to cell groups (**I**) and pseudotime (**II**); the arrow indicates the developmental direction. (**E**) Average PDUI distributions of differential APA events between hypoxic and normoxic cells. Only APA events detected in more than 20 cells in both groups were retained for analysis. (**F**) Kaplan–Meier curves for overall survival stratified by hypoxic cell abundance; significance was assessed by the log-rank test. (**G**) Frequency and strength of cell–cell interactions among hypoxia-related cancer cells, normoxia-related cancer cells, and other cell types, inferred using CellPhoneDB.

Moreover, analysis of the single-cell RNA-seq data [41] also revealed shortened 3’UTRs in hypoxic cells (Figure 2E), consistent with the preferential usage of proximal poly(A) sites observed under hypoxic conditions in both bulk RNA-seq and scRNA-seq datasets. In these data, normoxic and hypoxic cells were annotated with the markers provided in that study and analyzed using inferCNV [42] (Figure 2A,B and Figure S3A). This annotation was further validated using hypoxia-related gene set scores from MSigDB [32] (Figure 2C) and pseudo-time trajectory analysis (Figure 2D). Based on this framework, 2505 hypoxic cells, 2173 normoxic cells, and 3276 mixed cells were identified. Further, we observed that patients with a high abundance of hypoxic cells were significantly associated with poorer overall survival (Figure 2F). The results of cell–cell interaction analysis showed that the interaction intensity between hypoxic cells and other cell types, such as fibroblasts, was significantly higher than that between normoxic cells and other cell types (Figure 2G and Figure S3B). Transcriptional regulatory analysis revealed that transcription factors including *FOSL2*, *ETS2*, and *RXRA* were significantly enriched in hypoxic cells, while transcription factors such as *EHF*, *STAT1*, and *LTF* were significantly enriched in normoxic cells (Figure S3C). Previous studies have shown that *FOSL2* activates the FOSL2-ANXA1-FPR1/3 axis, thereby promoting macrophage polarization and facilitating tumor metastasis [53]. Overexpression of *STAT1* can inhibit the progression of glioma, as well as the expression of HIF-1α and VEGF-A, thereby exerting an antitumor effect [54].

### 3.2. Establishment and Validation of Hypoxia-Related Signature

**Figure 3:**
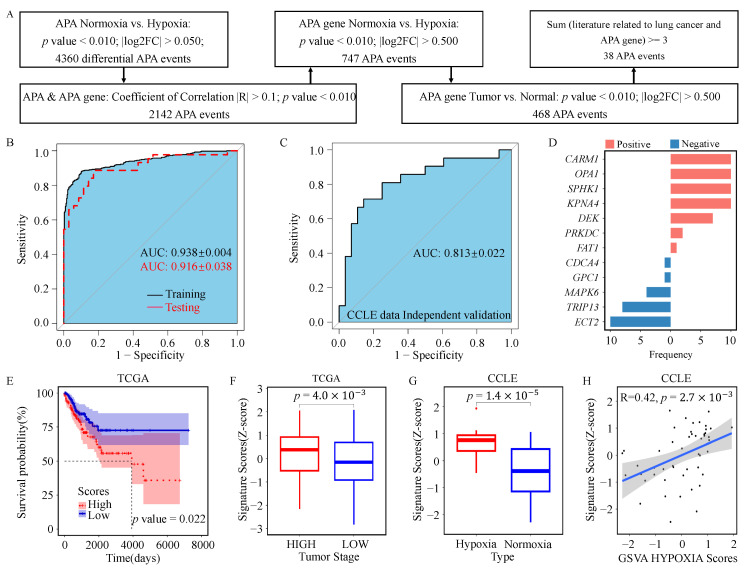
Identification and evaluation of hypoxia-related APA biomarkers in NSCLC. (**A**) Workflow for the identification of hypoxia-related APA events and construction of the HSS. Candidate APA events were first screened in TCGA and subsequently entered into logistic regression analysis for model construction, with CCLE used as an independent validation cohort. (**B**) Receiver operating characteristic (ROC) curves of logistic regression analysis based on 38 candidate APA events for classification of hypoxic and normoxic samples in the TCGA training set (black) and internal validation set (red), evaluated using ten-fold cross-validation. (**C**) ROC curves of the same logistic regression model in the independent CCLE validation cohort. (**D**) Selection frequencies of the 12 APA events retained in the final logistic regression model across ten-fold cross-validation. (**E**) Kaplan–Meier analysis of overall survival between high- and low-HSS patients in the TCGA cohort; significance was assessed by the log-rank test. (**F**) Comparison of HSS between high- and low-stage tumors by Student’s *t*-test. (**G**) Comparison of HSS between predefined hypoxic and normoxic cell lines by Student’s *t*-test. (**H**) Association between HSS and the HYPOXIA gene set score in CCLE cell lines, assessed using GSVA and Pearson correlation analysis.

The 5005 differential APA events identified in the TCGA dataset were further screened using the procedure shown in Figure 3A, resulting in 38 candidate APA events associated with tumor hypoxia. These candidate events occurred in genes with documented relevance to lung cancer (Table S1) and were therefore considered a biologically focused pool for subsequent model construction.

Using these 38 candidate APA events, logistic regression combined with ten-fold cross-validation achieved good discriminatory performance between hypoxic and normoxic states. The area under the receiver operating characteristic curve (AUC) was 0.938 ± 0.004 in the training cohort of the TCGA dataset, 0.916 ± 0.038 in the internal validation cohort of the TCGA dataset (Figure 3B), and 0.813 ± 0.023 in the independent CCLE validation cohort (Figure 3C).

During model construction, 12 APA events were retained in the final model (Figure 3D), and these events were used to construct the HSS (see Methods). Kaplan–Meier survival analysis showed that NSCLC patients with high HSS had significantly poorer overall survival than those with low HSS (Figure 3E). In addition, HSS was significantly higher in advanced-stage NSCLC tumors than in early-stage tumors (Figure 3F).

As expected from the feature-selection strategy, HSS differed significantly between the predefined hypoxic and normoxic groups in both patients and cell lines (Figure 3G and Figure S4B), and was positively correlated with the GSVA scores of the HYPOXIA, EPITHELIAL_MESENCHYMAL_TRANSITION, and GLYCOLYSIS gene sets from MSigDB [32] (Figure 3H and Figure S4C). These findings support the internal biological coherence of the constructed signature. At the spatial level, several hypoxia-related APA biomarkers, including *CARM1*, *ECT2*, *SPHK1*, *KPNA4*, *FAT1*, *GPC1*, and *TRIP13*, also showed supportive evidence across multiple tissue sections (Figure S5A,B). Together with the independent validation in the CCLE dataset and the observed associations with survival and tumor stage, these findings support the biological relevance, reproducibility, and potential translational significance of the HSS, including its possible utility for identifying clinically aggressive and hypoxia-associated NSCLC subgroups.

### 3.3. The Proximal Poly(A) Selection of *CARM1* Is a Candidate Biomarker of Hypoxic Tumors

Among the 12 APA event biomarkers, five APA events were found to be highly associated with tumor hypoxia across all ten different subsets of TCGA patients (Figure 3D). However, only one gene, coactivator-associated arginine methyltransferase 1 (*CARM1*), exhibited consistent associations across hypoxic status in patients and cell lines, advanced clinical stage tumors, and poorer survival outcomes. Previous studies have reported that *CARM1* is overexpressed in various cancers, promoting tumor invasion and metastasis, and interacts with *HIF1A* [19,55]. Therefore, we further focused on the APA events of this gene to explore its potential role in tumor hypoxia.

**Figure 4:**
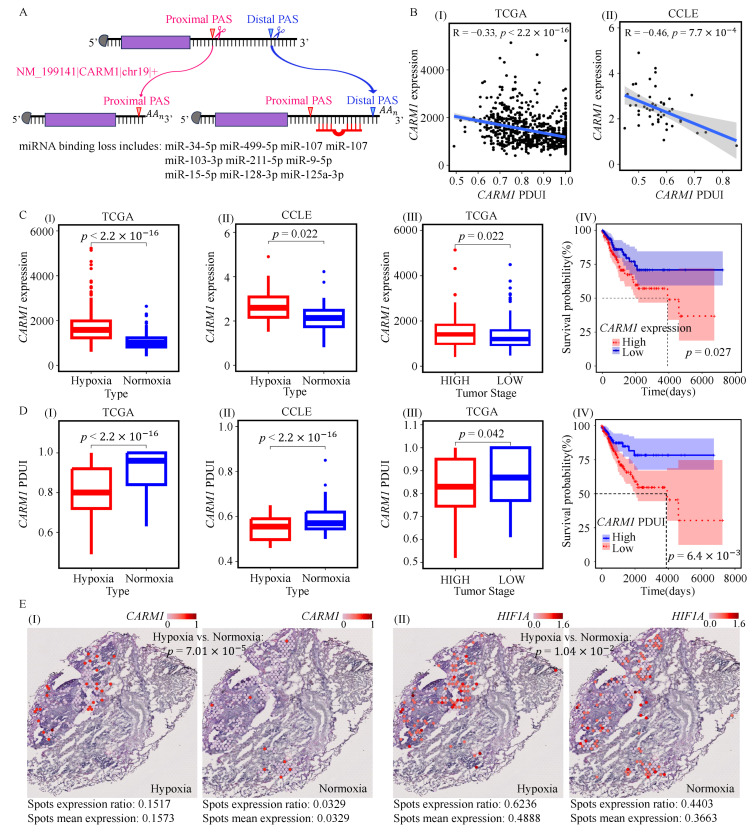
A potential mechanism for the association between APA events in *CARM1* and its own genes. (**A**) Schematic illustration showing that proximal poly(A) site usage of *CARM1* may shorten its 3′UTR and is predicted to reduce the number of putative miRNA-binding sites. (**B**) Associations between PDUI values of the *CARM1* APA event and *CARM1* expression in TCGA patients (**I**) and CCLE cell lines (**II**), assessed using Pearson correlation analysis. (**C**) Comparison of *CARM1* expression between hypoxic and normoxic patients (**I**), hypoxic and normoxic cell lines (**II**), low- and high-stage tumors (**III**), and low- and high-risk groups (**IV**). Group comparisons were performed by Student’s *t*-test, and survival-related comparisons were assessed by the log-rank test. (**D**) Comparison of PDUI values of the *CARM1* APA event between hypoxic and normoxic patients (**I**), hypoxic and normoxic cell lines (**II**), low- and high-stage tumors (**III**), and low- and high-risk groups (**IV**). Group comparisons were performed by Student’s *t*-test, and survival-related comparisons were assessed by the log-rank test. (**E**) Comparison of the expression ratio and average expression levels of *CARM1* and *HIF1A* between hypoxic and normoxic spatial spots by Student’s *t*-test.

For this gene, the proximal poly(A) site was more frequently selected in hypoxic samples. This pattern was associated with the predicted loss of miRNA binding sites (Figure 4A), including miR-125a-3p, which has been implicated in tumor hypoxia [56]. The full list of miRNAs predicted to lose binding sites upon APA-mediated shortening of *CARM1* is provided in Table S2. Consistently, proximal poly(A) site usage was associated with increased expression of *CARM1* (Figure 4B). In addition, higher expression levels of *CARM1* were observed in patients and cell lines with hypoxic features, advanced clinical stage, and poorer survival outcomes (Figure 4C). Correspondingly, lower PDUI values of *CARM1* were observed in these groups (Figure 4D). These findings suggest that proximal poly(A) site selection of *CARM1* may be associated with its upregulation in hypoxic NSCLC. At the spatial level, both the expression ratios and average expression levels of *CARM1* and *HIF1A* were higher in hypoxic spots compared to normoxic spots (Figure 4E).

**Figure 5:**
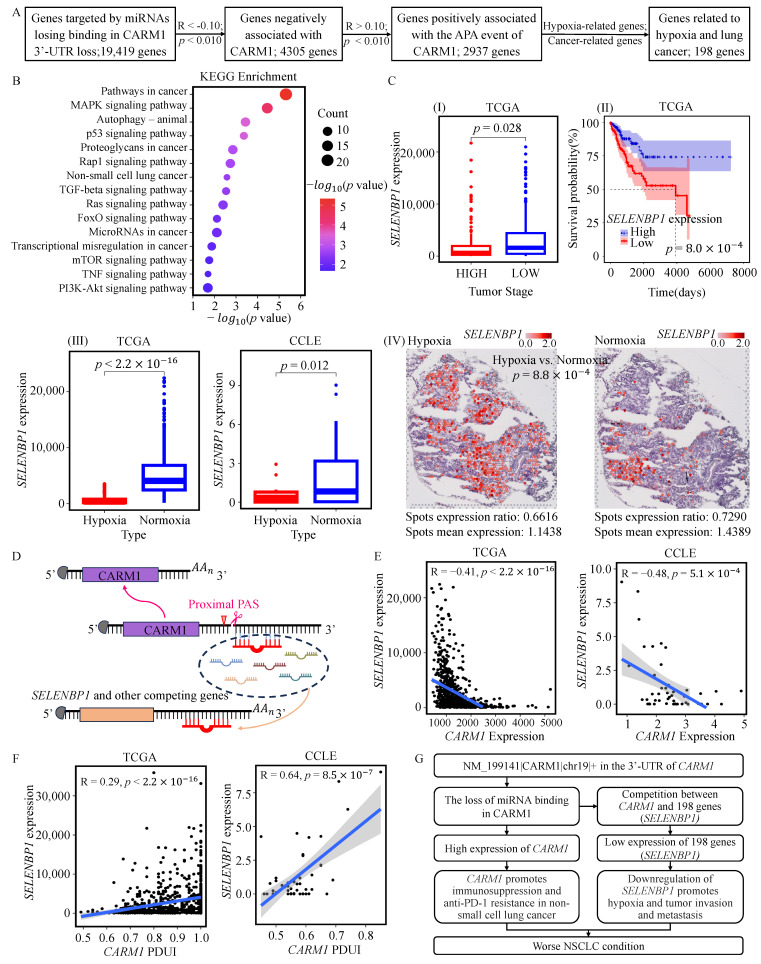
The APA event of *CARM1* may regulate other hypoxia-related genes with miRNA regulations. (**A**) The pipeline for identifying hypoxia related and lung cancer-related genes potentially associated with the APA event of *CARM1*. (**B**) Functional enrichment analysis of genes potentially associated with this APA event, performed using DAVID. (**C**) Comparison of *SELENBP1* expression across different clinical stages (**I**), survival-risk groups (**II**), hypoxic and normoxic patients (**III-left**), hypoxic and normoxic cell lines (**III-right**), and hypoxic and normoxic spatial spots (**IV**). Group comparisons were performed by Student’s *t*-test, and survival-related comparisons were assessed by the log-rank test. (**D**) Schematic illustration of a putative model in which proximal APA of *CARM1* may be associated with downstream regulation of genes such as *SELENBP1*. (**E**) Pearson correlations between *CARM1* and *SELENBP1* expression in TCGA patients (**left**) and CCLE cell lines (**right**). (**F**) Pearson correlations between PDUI values of the *CARM1* APA event and *SELENBP1* expression in TCGA patients (**left**) and CCLE cell lines (**right**). (**G**) Schematic summary of a hypothesis-generating model linking proximal APA of *CARM1* to hypoxia-related tumor progression in NSCLC.

We further explored the potential downstream effects associated with *CARM1* APA. The usage of the proximal poly(A) site may be associated with changes in miRNA availability, which in turn may influence the expression of other genes. These genes showed a significant negative correlation with *CARM1* expression and a significant positive correlation with the PDUI values of *CARM1* (Figure 5A). Among them, 198 genes were enriched in biological processes related to lung cancer and hypoxia (Figure 5B). One such gene, *SELENBP1*, a reported HIF-1 target and inhibitor of cancer progression [57], showed lower expression in samples with hypoxic status, higher tumor stage, and poorer survival outcomes (Figure 5C). Notably, among the miRNAs predicted to lose binding sites on *CARM1*, 262 of 892 (29.37%) were also predicted to target *SELENBP1* (Tables S2 and S3).

Consistently, *CARM1* expression was negatively correlated with *SELENBP1* expression (Figure 5E), while the PDUI values of *CARM1* were positively correlated with *SELENBP1* expression (Figure 5F). These observations provide supportive evidence for a potential regulatory relationship linking *CARM1* APA, miRNA-mediated regulation, and downstream gene expression. Taken together, these findings suggest a putative model in which proximal poly(A) site selection of *CARM1* may contribute to hypoxia-related tumor progression by modulating its expression and linking to altered regulation of downstream genes (Figure 5G).

### 3.4. The Proximal Poly(A) Site Selection of *CARM1* Is Associated with Cisplatin Drug Response in NSCLC

The above analysis revealed a possible mechanism linking proximal poly(A) site selection of *CARM1* to tumor hypoxia and cancer progression. Given that tumor hypoxia has been widely associated with resistance to anticancer therapies [58], we next explored whether proximal poly(A) site selection of *CARM1* was associated with cisplatin sensitivity.

**Figure 6:**
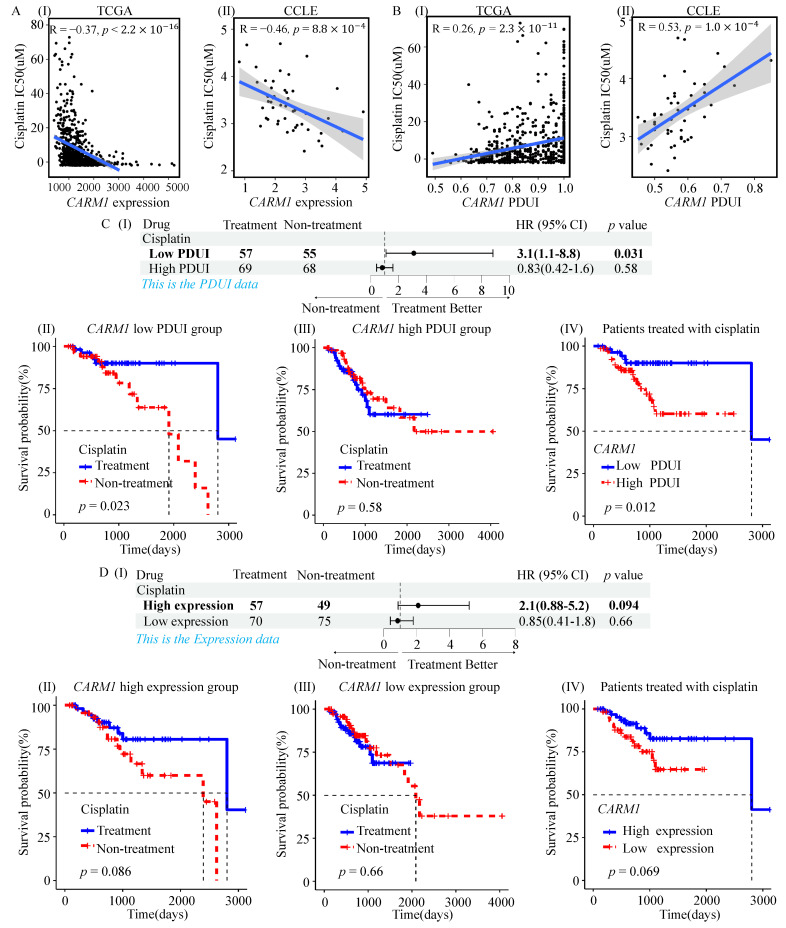
Drug sensitivity analysis of the APA event of *CARM1*. (**A**) Associations between predicted cisplatin sensitivity (IC50) and *CARM1* expression in TCGA patients (**I**) and CCLE cell lines (**II**), assessed using Pearson correlation analysis. (**B**) Associations between predicted cisplatin sensitivity (IC50) and PDUI values of the *CARM1* APA event in TCGA patients (**I**) and CCLE cell lines (**II**), assessed using Pearson correlation analysis. (**C**) Cox proportional hazards analysis (**top**) and Kaplan–Meier survival curves (**bottom**) comparing overall survival between high- and low-PDUI groups stratified by cisplatin treatment status in TCGA patients. PDUI groups were defined based on median values, and survival differences were assessed by the log-rank test. (**D**) Cox proportional hazards analysis (**top**) and Kaplan–Meier survival curves (**bottom**) comparing overall survival between high- and low-*CARM1* expression groups stratified by cisplatin treatment status in TCGA patients. Expression groups were defined based on median values, and survival differences were assessed by the log-rank test.

Based on CTD [39] and drug-treatment information available for TCGA patients, cisplatin was identified as a chemotherapeutic agent potentially relevant to *CARM1*. The predicted IC50 of cisplatin was significantly negatively correlated with *CARM1* expression in both patients and cell lines (Figure 6A), indicating that higher *CARM1* expression was associated with lower predicted IC50 values. In NSCLC, APA events were associated with the regulation of *CARM1* expression, and proximal poly(A) site selection of *CARM1* was significantly associated with increased predicted cisplatin sensitivity (Figure 6B). Consistent with these findings, analysis of NSCLC cell lines from the GDSC database showed that *CARM1* expression was significantly negatively correlated with cisplatin LN_IC50, whereas *CARM1* PDUI was significantly positively correlated with cisplatin LN_IC50. In addition, cisplatin LN_IC50 values were significantly higher in hypoxic cell lines than in normoxic cell lines (Figure S6).

We further examined retrospective clinical data from cisplatin-treated patients and found that survival patterns differed between patients with high and low PDUI values of the *CARM1* APA event (Figure 6C). In these retrospective analyses, patients with shortened 3’UTR of *CARM1* appeared to show more favorable survival patterns following cisplatin treatment, whereas this pattern was less evident in the high-PDUI group (Figure 6C-I–III). Survival analysis within cisplatin-treated patients (Figure 6C-IV) provided additional support for this association. Moreover, stratification based on this APA event showed stronger discriminatory ability in this retrospective setting than stratification based on *CARM1* expression alone (Figure 6D).

Taken together, these findings suggest that proximal poly(A) site selection of *CARM1* is associated with predicted cisplatin sensitivity and retrospective survival patterns in NSCLC, and may have potential value as a candidate biomarker for further investigation. However, as these analyses are based on predictive modeling and retrospective observational data, they should be interpreted as hypothesis-generating, and further validation will be required before any clinical application.

## 4. Discussion

Our study systematically explored the potential involvement of APA in NSCLC during the transition from normoxia to hypoxia, as well as changes in cellular composition and enhanced intercellular crosstalk within the tumor microenvironment. By extending the investigation of hypoxia in lung cancer from previously well-documented transcription factors [59,60], miRNAs [61,62,63,64], and proteins [65,66,67] to APA and the spatial dimensions, this study provides an additional perspective on the molecular features associated with hypoxic adaptation in NSCLC. Our findings suggest that APA may participate in the regulation of hypoxia-associated genes and be linked to tumor microenvironmental remodeling during tumor progression. These observations also support the potential relevance of APA in future therapeutic stratification strategies for patients with NSCLC [68,69].

At the spatial level, fibroblasts were more abundant in hypoxic spots than in normoxic spots, and this pattern was also observed in the surrounding cellular neighborhoods (Figure S7A,B). Spatial environment analysis using mistyR [70] further revealed significant spatial co-expression patterns between hypoxic cells and fibroblasts across the 1-spot, 7-spot, and 13-spot regions of the P16_T1 and P17_T1 slides (Figure S7C), suggesting a potential spatial association between these cell populations in the NSCLC microenvironment. Consistently, stLearn [71] analysis showed that 13 ligand-receptor pairs, including COL1A1_CD44, COL1A2_CD44, and FN1_CD44, had significantly higher interaction scores in hypoxic than in normoxic spots (Figure S7D). These ligand-receptor pairs were significantly enriched in cancer- and hypoxia-related pathways (Figure S7E), indicating that intercellular communication may be reshaped in hypoxic regions.

Additionally, by incorporating high-resolution spatial transcriptomics, we analyzed one NSCLC Visium HD slide. The proportions of eight cell types in each bin were estimated using spacexr [72], based on the cell types identified in single-cell data. Bins with a cancer cell abundance exceeding 20% were defined as cancer-related bins (Figure S8A). Hypoxia-related and normoxia-related bins were identified from these cancer bins based on CNV patterns (Figure S8B), combined with ssGSEA (Figure S8C), and visualized on the slide (Figure S8D). Based on this classification, we compared the average expression levels of tumor hypoxia biomarkers between hypoxic and normoxic bins. The tumor hypoxia biomarkers *SELENBP1*, *ECT2*, and *SPHK1* were validated in this slide (Figure S8E). Further, we conducted statistical analyses on the cellular composition within and around both hypoxic and normoxic bins. Compared to normoxic bins, the abundance of fibroblasts was significantly higher both within and around hypoxic bins (Figure S8F,G). The interaction strengths of receptor-ligand pairs, such as COL1A1_CD44, COL1A2_CD44, FN1_CD44, VIM_CD44, and COL1A1_ITGB1, were also significantly higher in hypoxic bins (Figure S8H). The analysis of Visium HD slides provided additional evidence for the findings of the tumor hypoxic microenvironment study.

These findings can also be interpreted in the context of several well-established hypoxia-related pathways. HIF-1 signaling is a central regulator of cellular adaptation to oxygen deprivation and coordinates transcriptional programs involved in angiogenesis, metabolism, survival, and invasion [73]. mTOR signaling has also been implicated in translational control and metabolic stress responses under hypoxic conditions [51]. Moreover, hypoxia is closely linked to EMT-associated phenotypes, stromal activation, extracellular matrix remodeling, and therapeutic resistance [4]. Therefore, the enrichment of fibroblasts and the increased interaction scores of extracellular matrix-related ligand–receptor pairs in hypoxic regions may reflect a microenvironmental context consistent with these established hypoxia-related programs in NSCLC, although the precise mechanistic relationships remain to be determined.

To strengthen the robustness of our findings, we validated key observations across multiple independent datasets and analytical strategies. For example, hypoxic and normoxic samples classified using different approaches showed broadly consistent patterns. Preferential usage of proximal poly(A) sites in hypoxic samples was consistently observed across NSCLC patient cohorts, cell lines, and single-cell datasets. Hypoxia-associated APA biomarkers were further supported in patient, cell line, Visium v2, and Visium HD datasets. In addition, the association between APA patterns and drug response was evaluated in both patient and cell line datasets, and predicted drug sensitivity was further compared with treatment-related clinical information from TCGA. Similarly, fibroblast enrichment and enhanced ligand-receptor interaction signals in hypoxic microenvironments were consistently detected in both Visium v2 and Visium HD datasets.

In parallel, the subgroup analyses further extended the personalized medicine context of our findings. Because the HSS was defined as a weighted linear combination of hypoxia-associated APA events, its consistent positive association with GSVA-derived hypoxia scores in both LUAD and LUSC suggests that this APA-based signature retains relevance across the two major NSCLC subtypes (Figure S9A). The HSS also showed associations with hypoxia-related phenotypes, poorer survival, and more advanced tumor stage, supporting its potential relevance as a candidate biomarker for identifying clinically aggressive and hypoxia-associated NSCLC subgroups (Figure S9B,C). We further observed that hypoxic tumors within both subtypes showed lower *CARM1* PDUI values, higher *CARM1* expression, and lower *SELENBP1* expression, supporting the consistency of the *CARM1*-related APA pattern in different histological backgrounds (Figure S9D). Moreover, HSS differed across clinically and molecularly relevant subgroups, including cisplatin treatment status and *KRAS* or *EGFR* mutation status (Figure S9E,F). Although these observations do not directly establish predictive utility for treatment response, they suggest that APA-associated hypoxia signatures may reflect biologically and clinically relevant heterogeneity in NSCLC and may be informative for future stratification studies. Because these findings are based primarily on retrospective and computational analyses, further validation will be required before any clinical application. Collectively, these cross-validation and subgroup analyses strengthen confidence in the robustness, biological relevance, and potential translational significance of our observations.

Among the identified APA-related candidates, proximal APA of *CARM1* emerged as a notable feature in NSCLC. Our analyses suggest that this event may be linked to tumor hypoxia, invasion, survival, and drug response, potentially through altered post-transcriptional regulation involving miRNAs. In addition to miRNAs, RNA-binding proteins (RBPs) may also participate in the regulatory consequences of APA (Figure S10A,B). For instance, proximal poly(A) site selection could reduce or abolish putative binding sites for RBPs such as IGF2BP2 (Figure S10C). Nevertheless, these observations are currently based solely on computational inference and should be interpreted cautiously. Further studies are required to clarify how APA-associated loss of miRNA and RBP binding sites influences *CARM1* regulation and related downstream phenotypes.

Despite these findings, several limitations warrant consideration. First, this study primarily relies on integrative bioinformatics and spatial transcriptomic analyses, without direct experimental validation in vitro or in vivo. Accordingly, the relationships among hypoxia-associated APA events, *CARM1* regulation, microenvironmental remodeling, and drug response should be interpreted as associative rather than causal. Second, the effects of APA on post-transcriptional regulation mediated by miRNAs and RBPs were inferred computationally and require further validation. This will involve experimental approaches such as luciferase reporter assays, isoform-specific perturbation, and miRNA gain- and loss-of-function studies. Third, although we performed cross-validation across multiple independent datasets, additional validation in prospective external cohorts and experimental models will be necessary to further establish the translational relevance of these findings. In addition, this study does not yet provide direct modeling of PD-1 immunotherapy response or machine-learning-based prediction using pharmacogenomics datasets, and these directions remain important topics for future work.

Overall, our study provides a multi-dimensional view of APA-associated molecular and spatial alterations in hypoxic NSCLC and highlights their potential relevance to tumor progression and therapeutic response. These findings offer a conceptual framework for future mechanistic and translational investigations of hypoxia-associated APA in NSCLC. A schematic summary of the proposed mechanisms is provided in Figure S13.

## 5. Conclusions

In summary, we systematically characterized hypoxia-associated APA alterations in NSCLC and explored their potential relevance to tumor progression, microenvironment remodeling, and therapeutic response. We observed widespread 3’UTR shortening in hypoxic tumors, suggesting a global shift toward proximal poly(A) site usage under hypoxic conditions. Based on these findings, we identified 12 hypoxia-associated APA events that exhibited robust performance in distinguishing hypoxic and normoxic states and were associated with patient prognosis. Among these, proximal poly(A) site selection of *CARM1* emerged as a representative candidate biomarker. This APA event was associated with increased *CARM1* expression and with expression changes in hypoxia-related genes such as *SELENBP1*. In parallel, single-cell and spatial transcriptomic analyses revealed increased fibroblast abundance and enhanced cell–cell interactions in hypoxic microenvironments. In addition, proximal poly(A) site selection of *CARM1* was associated with predicted cisplatin sensitivity, suggesting potential relevance for treatment stratification in NSCLC. Overall, our study highlights APA as a potentially important post-transcriptional regulatory layer in hypoxic NSCLC and provides candidate biomarkers and a framework for future mechanistic and translational studies.

## Data Availability

The datasets used in this study were obtained from TCGA, CCLE, and TC3A. The 10x Genomics Visium datasets for NSCLC slides were downloaded from BioStudies (accession: E-MTAB-13530). All data and code utilized in this study are publicly available at https://github.com/catly/NSCLC_Hypoxia/ (accessed on 15 April 2026).

## Supplementary Materials

The following supporting information can be downloaded at: https://www.mdpi.com/article/10.3390/genes17050505/s1, Figure S1. Workflow of hypoxia classification and consensus grouping using the NC and NMF methods. Figure S2. Additional analyses supporting the overview of APA in hypoxic lung cancer samples. Figure S3. Additional analyses of tumor hypoxia at the single-cell level. Figure S4. Additional analyses of hypoxia-related APA biomarker identification and HSS evaluation. Figure S5. Spatial transcriptomic analysis of tumor hypoxia in NSCLC. Figure S6. Additional validation of the association between *CARM1* related features, hypoxia status, and cisplatin response in NSCLC cell lines from the GDSC database. Figure S7. Additional spatial transcriptomic analyses of tumor hypoxia in NSCLC. Figure S8. Spatial transcriptomic analysis of hypoxic and normoxic bins in Visium HD slides. Figure S9. Additional subgroup analyses supporting the personalized medicine context of HSS and *CARM1*-related APA features in NSCLC. Figure S10. The effect of APA events on RNA-binding regulation of genes. Figure S11. Workflow for the integrated analysis of TCGA and CCLE RNA-seq data. Figure S12. The pipeline to analyze single-cell RNA-seq data. Figure S13. Schematic overview of hypoxia-associated APA regulation and its biological and clinical implications in NSCLC. Table S1. The genes associated with lung cancer according to previous studies. Table S2. Predicted miRNA-binding sites lost due to APA-mediated 3’UTR shortening of *CARM1*. Table S3. Overlapping miRNAs predicted to lose binding to *CARM1* and to target *SELENBP1*.

## Abbreviations

The following abbreviations are used in this manuscript:

3’UTR	3’ untranslated regions
APA	Alternative polyadenylation
AUC	Area under the receiver operating characteristic curve
BP	Biological process
CCLE	Cancer Cell Line Encyclopedia
CTD	Comparative Toxicogenomics Database
EMT	Epithelial–mesenchymal transition
GSVA	Gene Set Variation Analysis
HR	Hazard ratio
HSS	Hypoxia-Signature Scores
IC50	Half-maximal inhibitory concentration
KEGG	Kyoto Encyclopedia of Genes and Genomes
MSigDB	Molecular Signatures Database
NMF	Non-negative Matrix Factorization
NSCLC	Non-small cell lung cancer
PAS	Poly(A) site
PCA	Principal component analysis
PDUI	Percentage of distal poly(A) site usage index
RBP	RNA-binding protein
scRNA-seq	single-cell RNA sequencing
ssGSEA	Single-sample gene set enrichment analysis
TC3A	The Cancer 3’UTR Altas
TCGA	The Cancer Genome Atlas
UMAP	Uniform Manifold Approximation and Projection
UMI	Unique Molecular Identifier
