# Supplementary material for: Hypoxia-Associated Alternative Polyadenylation of CARM1 and Tumor Microenvironment Alterations in Non-Small Cell Lung Cancer
Source: Genes (Basel). 2026 Apr 24;17(5):505. doi: 10.3390/genes17050505 (PMC13205813; doi:10.3390/genes17050505)
Supplement: Supplementary file 1 [file genes-17-00505-s001.zip › SupplementaryFigures.pdf]

Supplemental materials

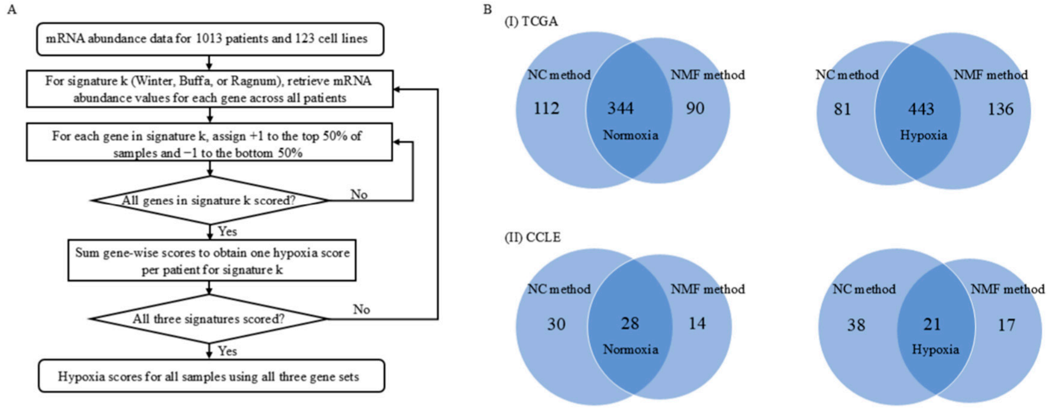

Figure S1. Workflow of hypoxia classification and consensus grouping using the NC and NMF methods. A. Schematic illustration of the NC-based hypoxia scoring workflow. mRNA abundance profiles from 1013 NSCLC patients and 123 NSCLC cell lines were analyzed using the Winter, Buffa, and Ragnum hypoxia gene signatures. For each signature, each gene was scored across all samples, and the resulting gene-wise scores were then summed to generate a hypoxia score for each sample. B. Consensus classification of samples into normoxic and hypoxic groups based on the NC and NMF methods in the TCGA and CCLE datasets. Venn diagrams show the overlap between the two methods for normoxic and hypoxic assignments. Samples consistently classified by both methods were assigned to the normoxic or hypoxic group, whereas the remaining samples were categorized as mixed.

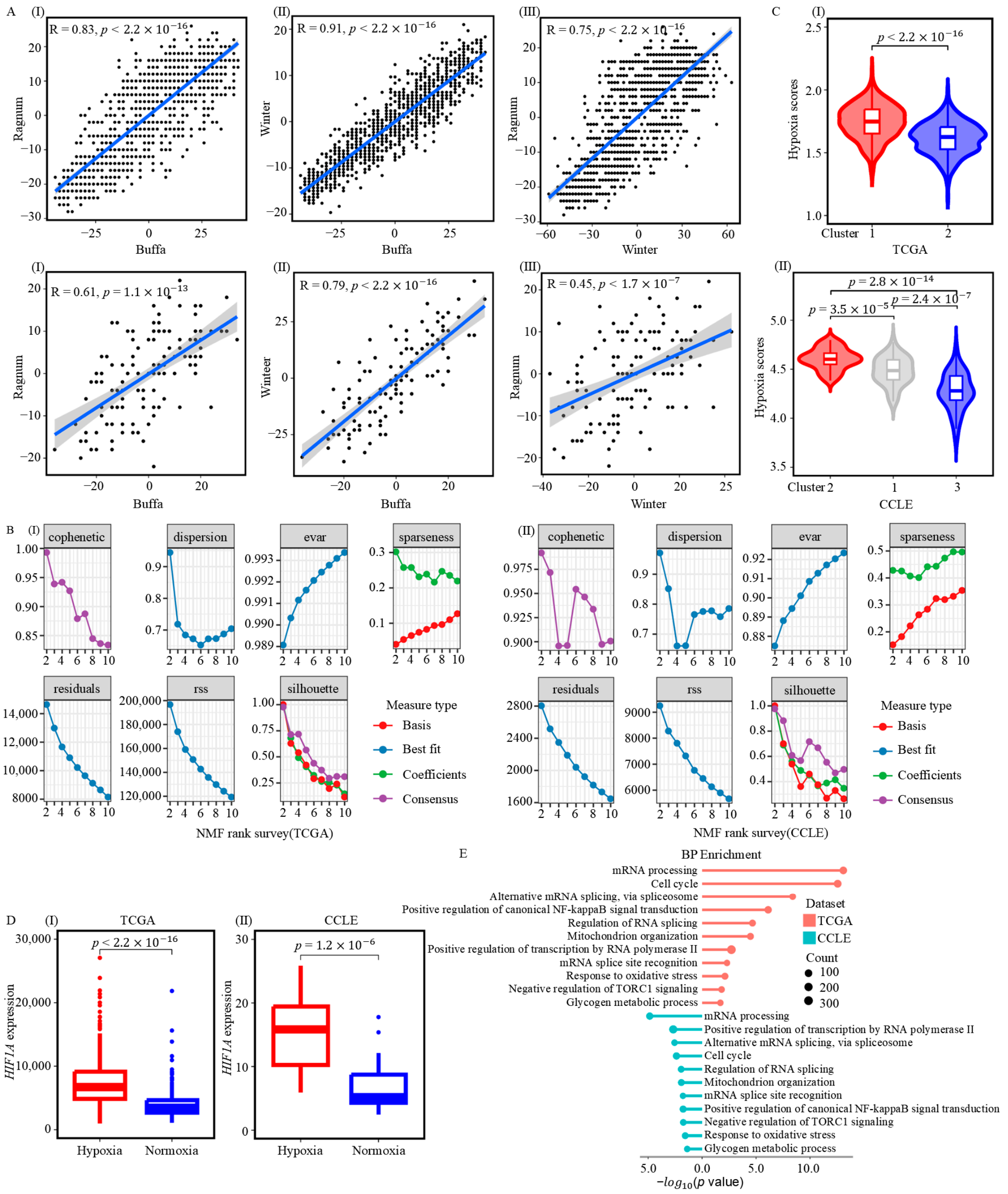

Figure S2. Additional analyses supporting the overview of APA in hypoxic lung cancer samples. A. Correlation between hypoxia scores calculated using the Buffa, Winter, and Ragnum gene signatures in TCGA (top) and CCLE (bottom) samples, assessed using Pearson correlation analysis. B. Determination of the optimal number of clusters in TCGA patients (left) and CCLE cell lines (right) using NMF, based on the cophenetic correlation coefficient. The selected rank corresponds to the point just before the largest decrease in the cophenetic coefficient. C. Comparison of HYPOXIA gene set scores among different clustering groups was performed using the Student's t-test. D. Comparison of HIF1A expression between normoxic and hypoxic groups in TCGA patients (left) and CCLE cell lines (right) using the Student's t-test. E. Enriched biological process (BP) terms of genes associated with differential APA events in TCGA

patients (top) and CCLE cell lines (bottom), identified using the DAVID tool with appropriate multiple testing correction.

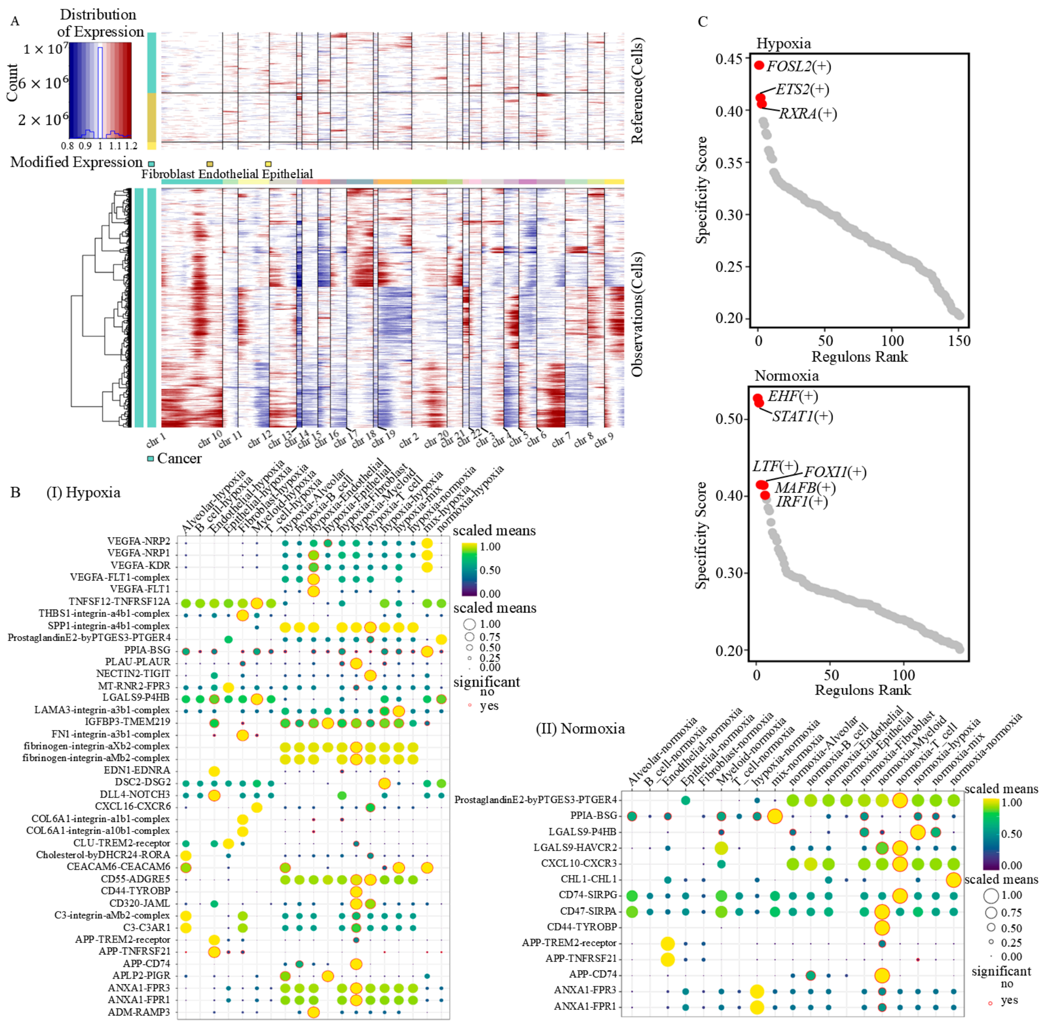

Figure S3. Additional analyses of tumor hypoxia at the single-cell level. A. Identification of hypoxic and normoxic malignant cells using InferCNV analysis. Copy number variation (CNV) patterns were inferred in tumor cells using fibroblasts, epithelial cells, and endothelial cells as reference populations. B. Predicted ligand–receptor interactions between hypoxia-associated cancer cells, normoxia-associated cancer cells, and other cell types were analyzed using CellPhoneDB. Interaction strength and frequency are shown. C. Expression patterns of transcription factors significantly enriched in hypoxia-associated and normoxia-associated cancer cells were identified using pySCENIC analysis.

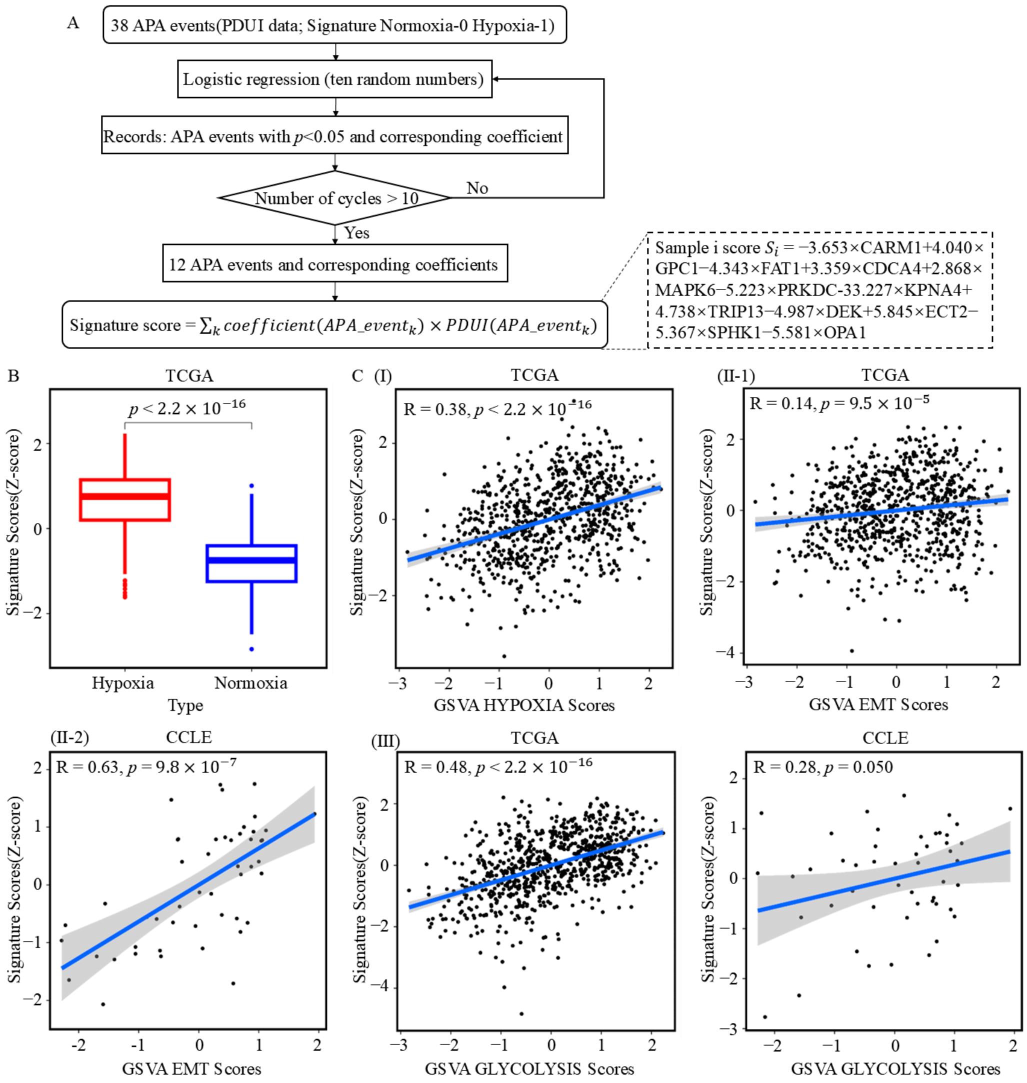

Figure S4. Additional analyses of hypoxia-related APA biomarker identification and HSS evaluation. A. Detailed workflow for the selection of 12 APA events and construction of the HSS using logistic regression analysis, with model training and validation performed as described in the Methods. B. Comparison of HSS between predefined normoxic and hypoxic patients in the TCGA cohort using Student's t-test. C. Correlation between HSS and three hypoxia-related gene set scores in TCGA patients and CCLE cell lines, calculated using GSVA and assessed by Pearson correlation analysis.

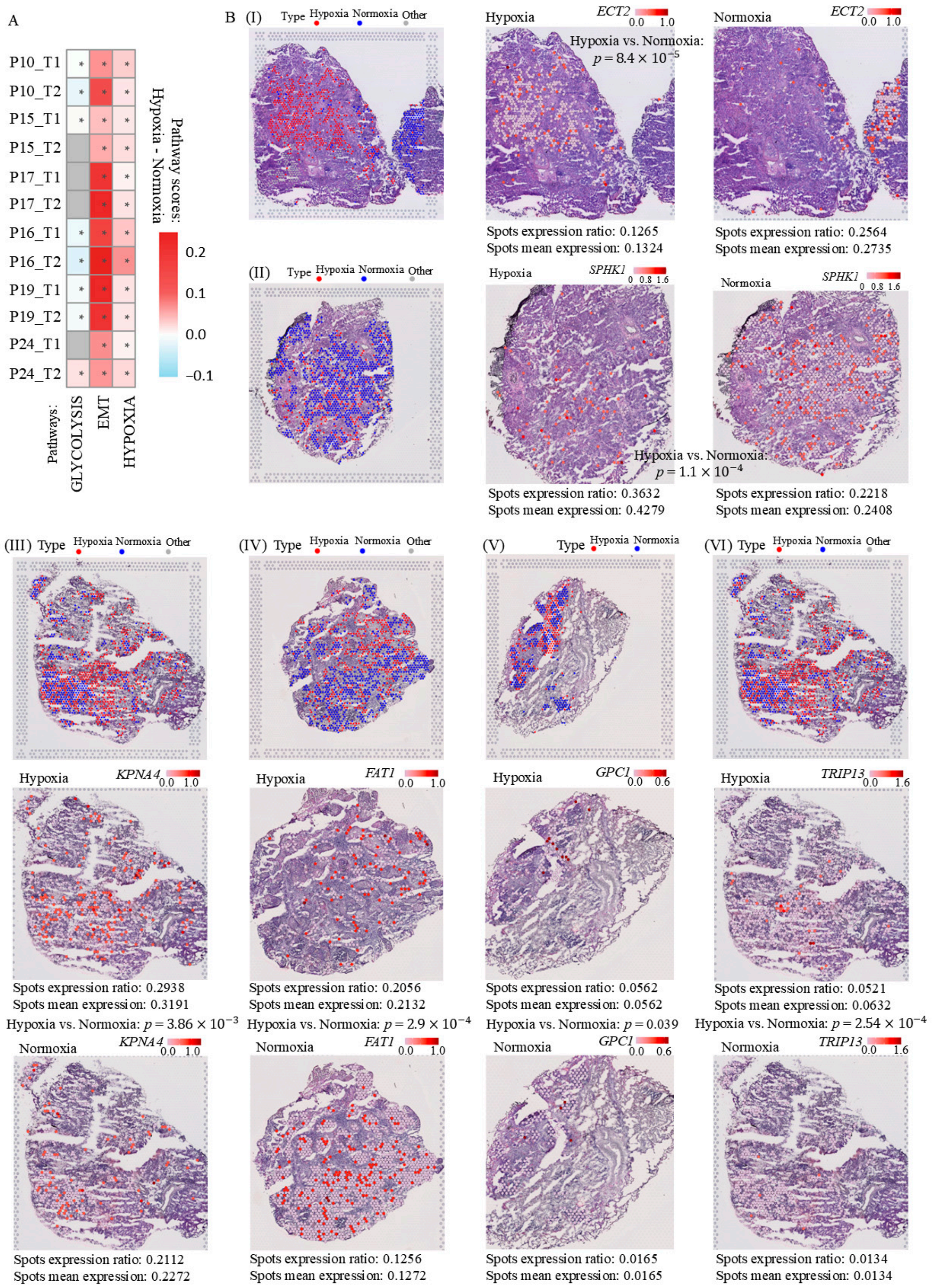

Figure S5. Spatial transcriptomic analysis of tumor hypoxia in NSCLC. A. Comparison of two hypoxia-related gene set scores between predefined hypoxic and normoxic spatial spots using the Wilcoxon rank-sum test. B. Comparison of the expression levels of hypoxia-related biomarkers between hypoxic and normoxic spatial spots using the Student's t-test. (*, $p < 0.01$)

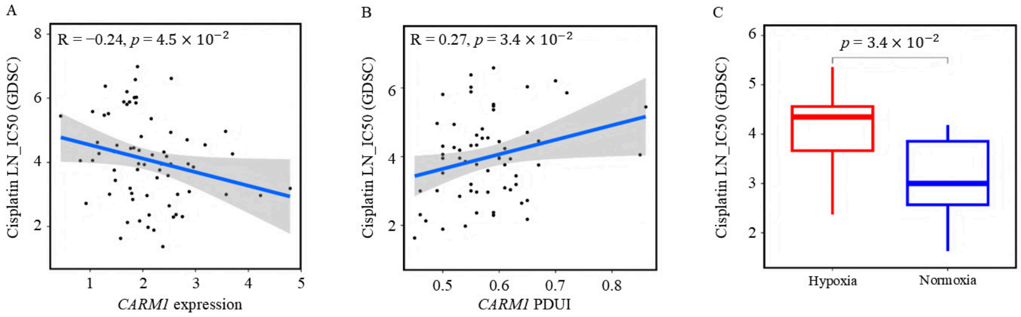

Figure S6. Additional validation of the association between *CARM1*-related features, hypoxia status, and cisplatin response in NSCLC cell lines from the GDSC database. A. Correlation between *CARM1* expression and cisplatin LN_IC50 in NSCLC cell lines, assessed by Pearson correlation analysis. B. Correlation between *CARM1* PDUI and cisplatin LN_IC50 in NSCLC cell lines, assessed by Pearson correlation analysis. C. Comparison of cisplatin LN_IC50 between predefined hypoxic and normoxic cell lines using Student's t-test.

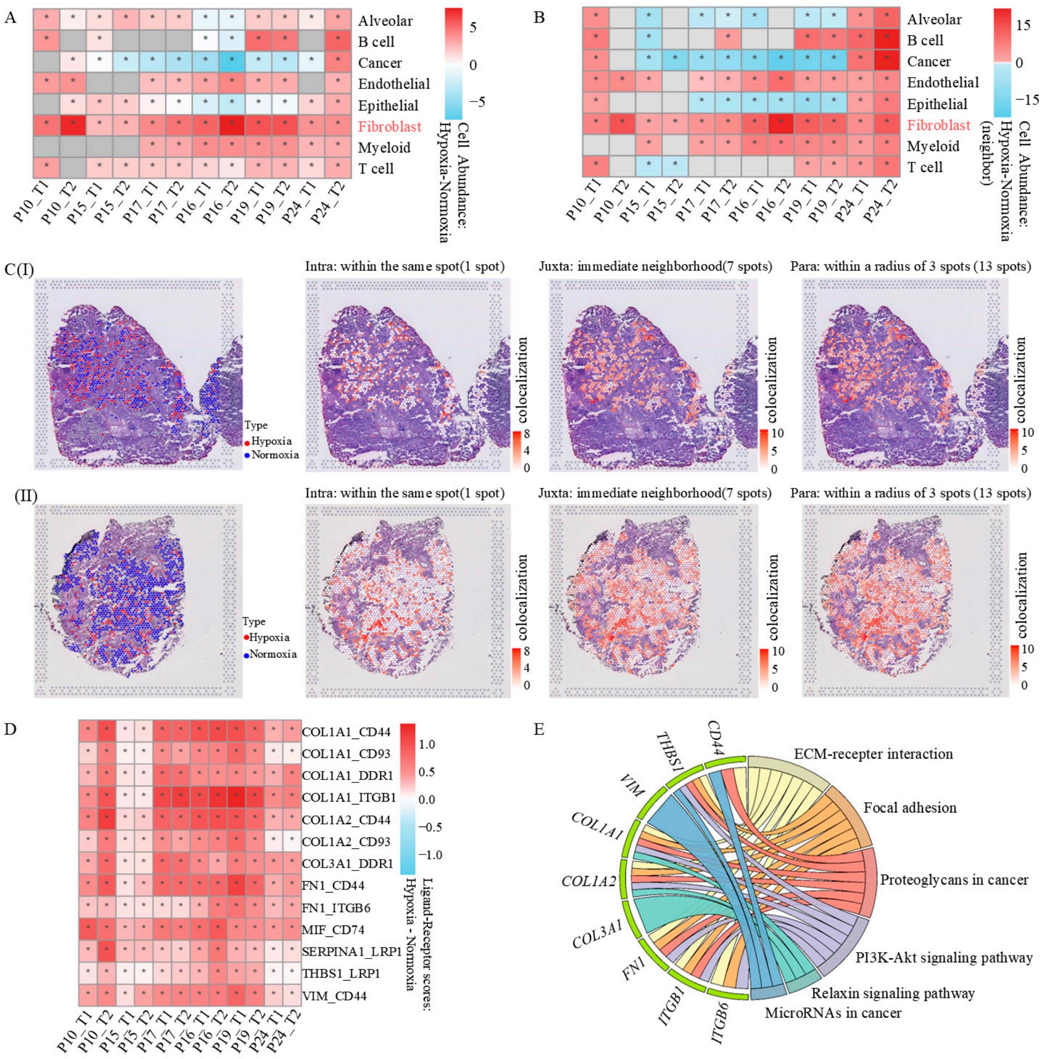

Figure S7. Additional spatial transcriptomic analyses of tumor hypoxia in NSCLC. A. Comparison of proportions of different cell types between hypoxic and normoxic spatial spots. B. Comparison of cell-type proportions in the tumor microenvironment (defined as the six immediate neighboring spots of each focal spatial spot) between hypoxic and normoxic regions. C. Colocalization analysis showing increased spatial co-occurrence between hypoxia-associated cancer cells and fibroblasts in representative slides (P16_T1 and P17_T1). D. Comparison of ligand-receptor interaction scores between hypoxic and normoxic spots identified 13 ligand–receptor pairs with significantly higher interaction activity in hypoxic regions, assessed via the Wilcoxon rank-sum test. E. KEGG pathway enrichment of ligand–receptor-associated genes identified using the DAVID tool with appropriate multiple testing correction. (*, $p < 0.01$)

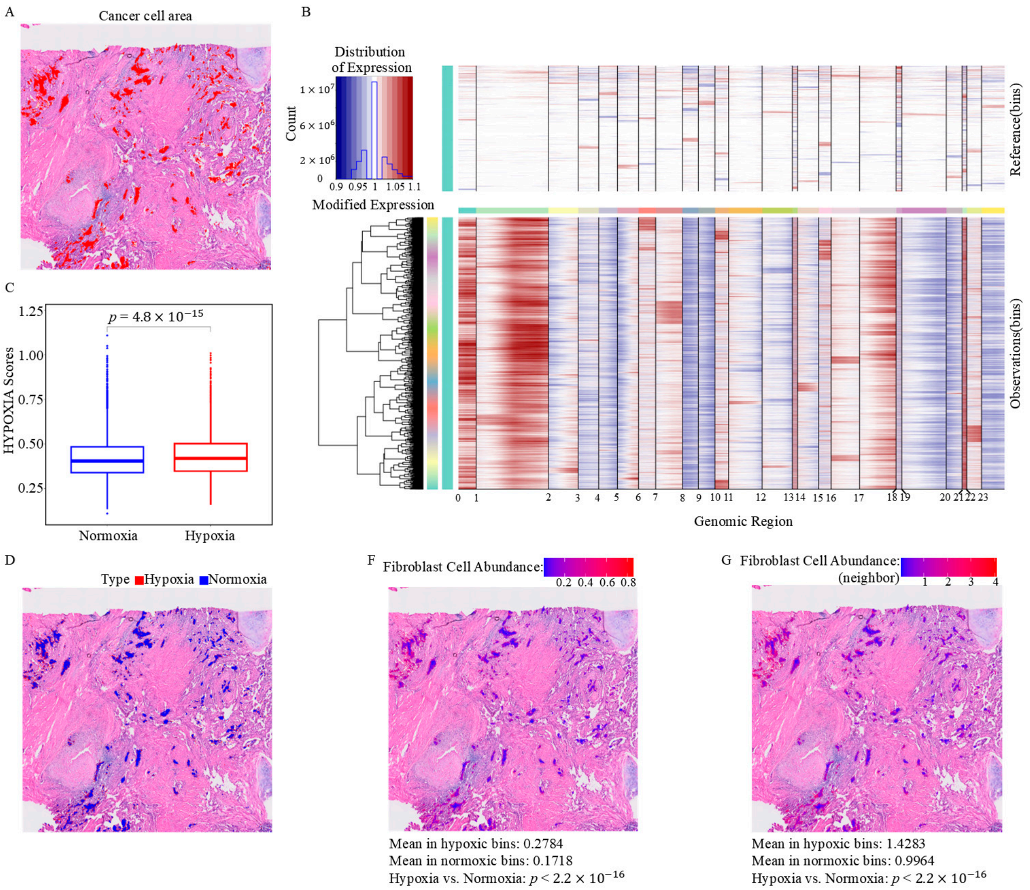

E

Genes	Expression (Normoxia)	Expression (Hypoxia)	Diff	p_value (t-test)
SPHK1	0.02040174	0.02841303	0.00801129	1.2×10^{-3}
ECT2	0.03440499	0.02252252	−0.01188247	1.0×10^{-6}
SELENBP1	0.18136047	0.08090783	−0.1004526	$< 2.2 \times 10^{-16}$

H

Ligand-receptor	Mean(Normoxia)	Mean(Hypoxia)	Diff	p_adjust
COL1A1_CD44	0.898994581	1.024292987	0.125298406	1.03×10^{-20}
COL1A2_CD44	0.573421616	0.698495023	0.125073407	5.41×10^{-36}
FN1_CD44	0.300488465	0.4016419	0.101153435	2.26×10^{-42}
FN1_PLAUR	0.167996619	0.241845948	0.073849329	8.79×10^{-37}
VIM_CD44	0.270191857	0.326129803	0.055937946	2.09×10^{-18}
COL1A1_ITGB1	0.327866973	0.383320879	0.055453906	1.17×10^{-8}
COL1A2_ITGB1	0.205844703	0.259079753	0.05323505	1.03×10^{-14}

Figure S8. Spatial transcriptomic analysis of hypoxic and normoxic bins in Visium HD slides. A. Distribution of cancer cell abundance across spatial bins within tumor regions. B. Identification of hypoxic and normoxic tumor bins using InferCNV analysis based on inferred copy number variation (CNV) profiles. C. Comparison of HYPOXIA gene set scores between hypoxic and normoxic bins using the Wilcoxon rank-sum test. D. Spatial distribution of hypoxic and normoxic bins within tumor regions. E. Comparison of the expression levels of hypoxia-related biomarkers between hypoxic and normoxic bins using the Student's t-test. F. Comparison of proportions of different cell types between hypoxic and normoxic bins. G. Comparison of cell-type proportions in the tumor microenvironment (defined as adjacent bins) between hypoxic and normoxic regions. H. Comparison of ligand–receptor interaction scores between hypoxic and normoxic bins, identifying 7 pairs with significantly higher interaction activity in hypoxic bins, assessed using the Wilcoxon rank-sum test.

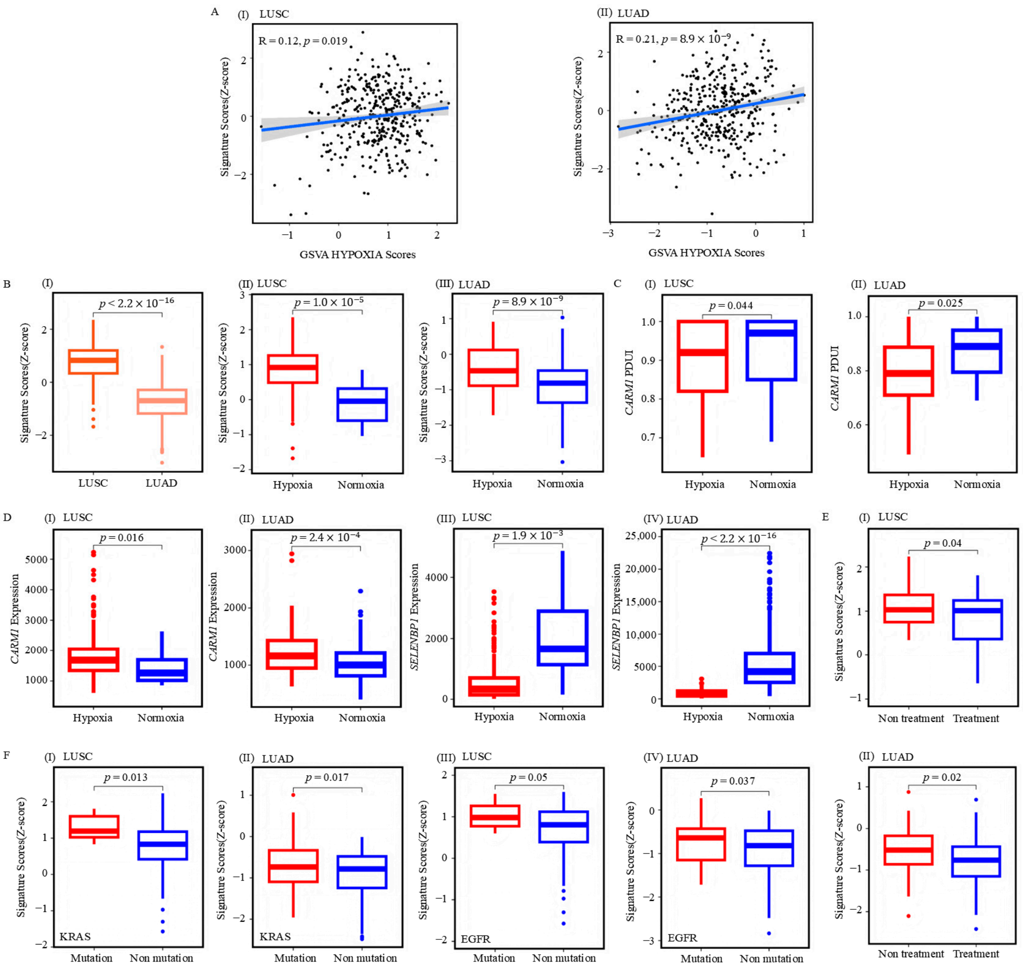

Figure S9. Additional subgroup analyses supporting the personalized medicine context of HSS and *CARM1*-related APA features in NSCLC. A. Correlation between HSS and GSVA-derived hypoxia scores in LUSC and LUAD, assessed using Pearson correlation analysis. B. Comparison of HSS between LUSC and LUAD, and between hypoxic and normoxic tumors within each subtype, using the Student's t-test. C. Comparison of *CARM1* PDUI values between hypoxic and normoxic tumors in LUSC and LUAD using the Student's t-test. D. Comparison of *CARM1* and *SELENBP1* expression levels between hypoxic and normoxic tumors in LUSC and LUAD using the Student's t-test. E. Comparison of HSS between patients with and without cisplatin treatment in LUSC and LUAD using the Student's t-test. F. Comparison of HSS between mutant and non-mutant groups for *KRAS* and *EGFR* in LUSC and LUAD using the Student's t-test. LUSC, Lung Squamous Cell Carcinoma; LUAD, Lung Adenocarcinoma. $p < 0.05$ was considered statistically significant.

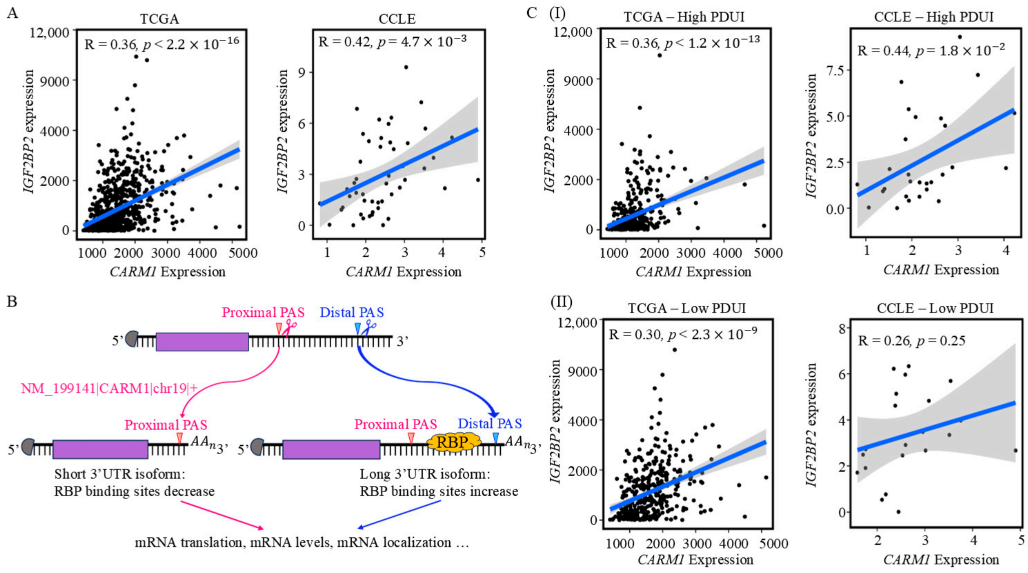

Figure S10. The effect of APA events on RNA-binding regulation of genes. A. Correlation between the RNA-binding protein *IGF2BP2* and *CARM1* expression in TCGA patients (left) and CCLE cell lines (right), assessed by Pearson correlation analysis. B. Schematic illustration showing that proximal poly(A) site usage may reduce the availability of putative RBP-binding sites. C. Correlation between *IGF2BP2* and *CARM1* expression in samples stratified by distal poly(A) site usage (top) and proximal poly(A) site usage (bottom), assessed by Pearson correlation analysis.

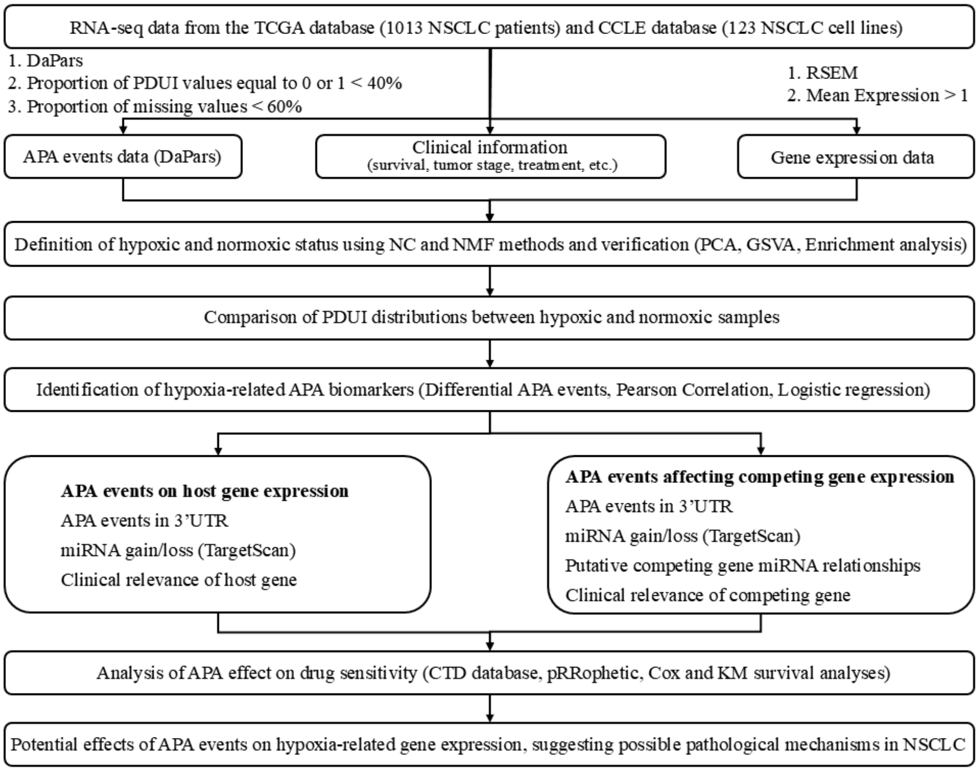

Figure S11. Workflow for the integrated analysis of TCGA and CCLE RNA-seq data.

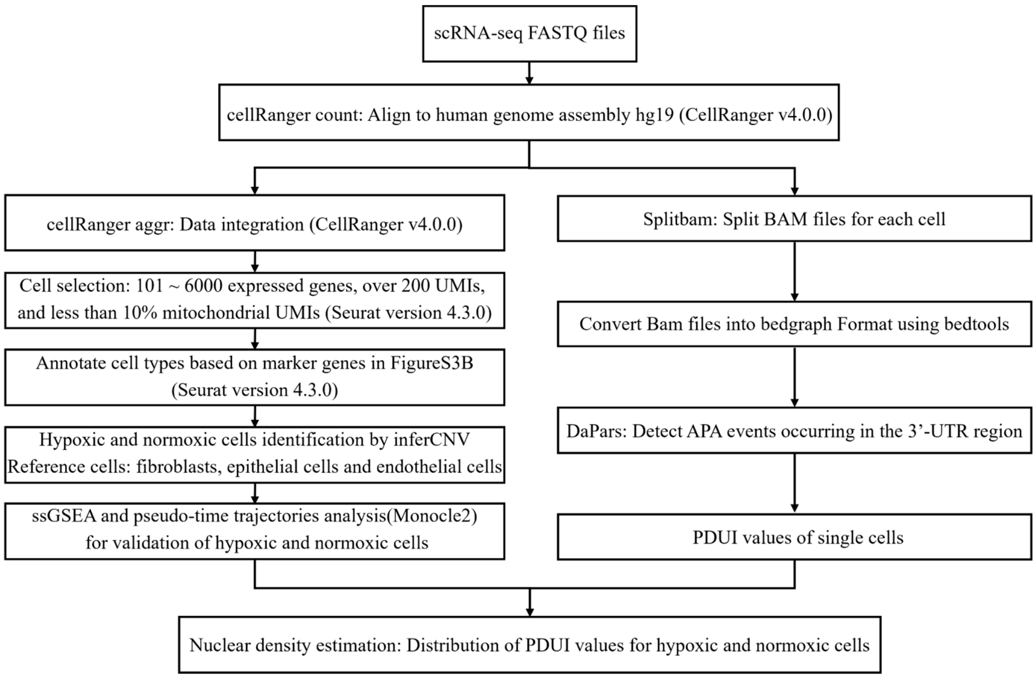

Figure S12. The pipeline to analyze single-cell RNA-seq data.

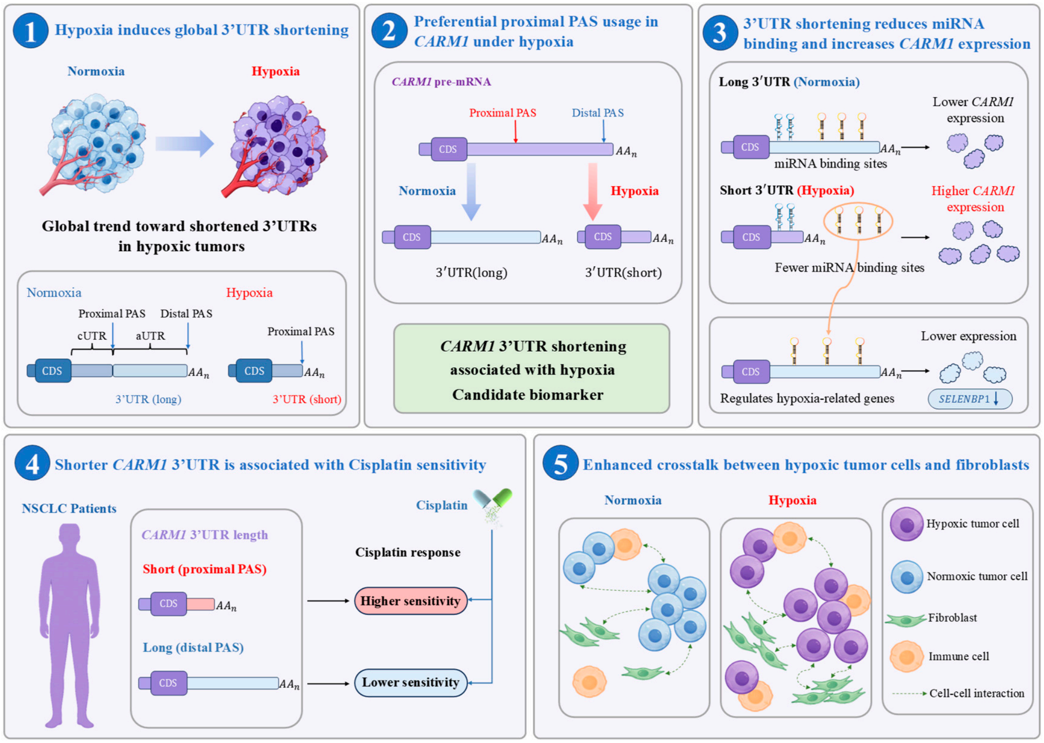

Figure S13. Schematic overview of hypoxia-associated APA regulation and its biological and clinical implications in NSCLC. Under hypoxic conditions, widespread 3'UTR shortening is observed, reflecting a global shift toward proximal poly(A) site usage. This process reduces miRNA binding sites and contributes to increased expression of target genes. *CARM1* is highlighted as a representative APA-regulated gene, where proximal poly(A) site selection is associated with elevated expression and may influence downstream hypoxia-related pathways, including genes such as *SELENBP1*. In parallel, hypoxia is associated with increased intercellular communication between tumor cells and fibroblasts in the tumor microenvironment. In addition, APA events in *CARM1* are associated with predicted cisplatin sensitivity, suggesting their potential relevance to therapeutic response in NSCLC. aUTR, alternative untranslated region; cUTR, constitutive untranslated region; PAS, poly(A) signal.
